# A systematic review of couple- or household-targeted interventions for smoking cessation in pregnancy

**DOI:** 10.1186/s12889-026-27484-0

**Published:** 2026-05-08

**Authors:** Sadie Mullin, Christy Burden, Kate Grant, Abi Merriel, Anna Davies

**Affiliations:** 1 Academic Women’s Health Unit, Translational Health Sciences, Bristol Medical School, University of Bristol, Bristol, BS8 1QU UK; 2 North Bristol NHS Trust, Southmead Hospital, Southmead Road, Bristol, BS10 5NB UK; 3 University Hospitals Bristol and Weston NHS Foundation Trust, Marlborough Street, Bristol, BS1 3NU UK; 4 South Gloucestershire Council, Public Health and Wellbeing Division, Badminton Road, Yate, Bristol, BS37 5AF UK; 5 Department of Women’s and Children’s Health, University of Liverpool, Liverpool, L7 8TX UK

**Keywords:** Smoking cessation, Pregnancy, Partners, Couples-based, Household, Nicotine-replacement, Behavioural support

## Abstract

**Background:**

Tobacco smoking is a major modifiable risk for poor pregnancy outcomes. Pregnant women whose partner smokes are six times more likely to continue smoking. Typically, interventions target pregnant mothers or smoking fathers to reduce second-hand smoke exposure. Evidence reviews advocate for couple-targeted smoking cessation interventions, however no recent review has evaluated the effectiveness of interventions targeting both pregnant women and their partner/household member. We aimed to systematically review and evaluate the effectiveness of smoking cessation interventions targeting the couple/household where both parties smoke for antenatal smoking cessation in pregnant women.

**Methods:**

We searched 8 databases (MEDLINE, Embase, Emcare, AMED, BNI, CINAHL, PsycINFO, Cochrane Register of Controlled Trials) for randomised controlled trials (RCTs), controlled non-randomised studies and before-and-after studies, meeting PICO criteria: Population: pregnant woman and her partner/household member/s who are tobacco smokers antenatally. Intervention: Tobacco smoking cessation intervention targeting a couple/household. Control: Intervention targeting woman only, usual care, historical control group. Outcome/s: Objectively assessed or self-reported antenatal maternal smoking cessation. Studies were systematically selected for inclusion and data were extracted by two researchers. Meta-analysis was not undertaken due to clinical and methodological heterogeneity, including differing intervention types, outcome measures, cut-offs used to define cessation and follow-up timepoints. Data were narratively synthesised.

**Results:**

Six studies were included: 4 RCTs, 1 non-randomised comparative study and 1 before-and-after study. Interventions were poorly described, variable in content across studies, and differed in content between women and partners/household members within studies. They included behavioural support (*n* = 6 women, *n* = 4 partners), written or self-help materials (*n* = 4 women, *n* = 6 partners), nicotine replacement therapy (*n* = 1 women, *n* = 1 partners), demonstration of smoking effects on the fetal heart (*n* = 1 women) and incentives (*n* = 1 women). Three studies directly targeted partners and three targeted them indirectly via the woman. Only one study compared targeting the couple versus the woman only. Varied subjective and objective cessation measures were assessed. Quit definition, measurement timepoint, and whether partner cessation was evaluated varied. Two RCTs, one non-randomised controlled and one before-and-after study reported that their intervention positively impacted smoking cessation for women compared with usual care. One RCT reported increased cessation for intervention versus control for partners.

**Conclusions:**

Few up-to-date studies have evaluated smoking cessation interventions targeting couples/households who smoke during pregnancy. Interventions rarely directly target partners. Interventions directly targeting the woman and her partner or smoking household members, using up-to-date interventions (e.g., nicotine replacement therapy, vapes, behavioural support) are yet to be assessed in high quality studies and more evidence in this field is needed. Standardised outcomes for both women and partners are needed to evaluate efficacy, identify the active components of interventions and facilitate evidence syntheses.

**Supplementary Information:**

The online version contains supplementary material available at 10.1186/s12889-026-27484-0.

## Introduction

Cigarette smoking is the largest modifiable risk factor for poor maternal and perinatal outcomes in pregnancy, including intra-uterine growth restriction, pre-term labour and stillbirth, and postnatal impacts include sudden infant death syndrome (SIDS) and childhood asthma [1, 2]. A US study examining over 13 million maternal infant-pairs identified a dose–response relationship between maternal cigarette smoking in pregnancy and all-cause, all-specific infant death [3].

The prevalence of smoking in pregnancy varies internationally with global rates estimated to be 1.7% [4]. The highest rates of smoking are demonstrated in Europe at 8.1%, and estimates indicate that 30.6% of European women who smoke daily continue to do so in pregnancy [4]. In the UK, the NHS England *‘Saving Babies’ Lives’* care bundle was introduced to halve perinatal mortality by 2025. This included directly targeting smoking in pregnancy via introduction of regular carbon monoxide (CO) testing and an opt-out referral to a tobacco dependence advisor for behavioural support and nicotine replacement therapy (NRT) [5]. England’s Smoking at Time of Delivery rate (SATOD) has been slow to decrease, with a rate of 5.6% reported in January 2025 compared with 10.4% in 2016 [6].

An important predictor of cessation during pregnancy is household smoking. Both qualitative [7] and quantitative studies indicate that women living with a smoker are less likely to quit during pregnancy and remain abstinent postnatally [8, 9]. In the UK, data are not reported in national online datasets (NHS Digital) as to whether women are living with a smoking partner. Data from an observational study of women in Spain indicated a high smoking prevalence in the partners of women who were smoking in early pregnancy at 37% [10]. Research has demonstrated that a woman’s failure to quit and reduce the number of cigarettes smoked in pregnancy was independently associated with her partner failing to stop smoking and with women’s close social contacts continuing to smoke [11]. Importantly, evidence suggests that pregnancy may provide a unique window of motivation and opportunity for women and their partners, and potentially the wider family, to change their smoking behaviour, since they may be motivated to protect the baby from adverse smoking impacts [12].

Therefore, interventions targeting household smoking cessation may be important for facilitating a successful maternal antenatal quit attempt. Despite this, evidence reviews to identify effective interventions have typically focused on interventions to support the mother [13, 14] and interventions to support cessation in fathers where a non-smoking mother is exposed to second-hand tobacco smoke at home [15, 16]. A more recent Cochrane review in 2018 of interventions for non-pregnant smokers concluded that interventions that aim to enhance partner support had no impact on increasing long‐term abstinence from smoking [17]. This was because most interventions tested were unable to increase the partner support received by smokers. Therefore, the authors could not conclude whether no effect on cessation was because partner‐support interventions do not work to increase cessation in smokers, or because the interventions tested were not able to increase the level of support from partners to smokers trying to quit. A systematic review conducted in 2012 explored effectiveness of interventions to enhance partner support for pregnant and postpartum women’s smoking reduction or cessation and treatments for partners themselves [18]. Nine studies were identified, of which three targeted both the woman and her partner directly and two addressed women as a route to influencing male partners. The limited number of studies meeting this review’s inclusion criteria suggest a need for further, up-to-date exploration of the evidence base for couples or household-targeted smoking cessation interventions in pregnancy.

We aimed to undertake a systematic review to evaluate the effectiveness of smoking cessation interventions targeting pregnant women who smoke along with their smoking partners/household members on antenatal smoking cessation.

## Method

### Funding

This work was funded by an NIHR academic clinical fellowship.

### Registration

This review was registered on the PROSPERO database (CRD42022196297) on 22nd December 2022 [19]. No protocol for the review was prepared.

### Eligibility criteria

Eligibility criteria for the review were defined relating to the population, intervention, control group, outcome and study design criteria (PICOS) [20].

#### Population

We included studies in which a pregnant woman:

- who self-identified or was identified as a cigarette smoker during the antenatal period was offered a smoking cessation intervention.
- AND her partner/family/household member was also a smoker and was also offered an antenatal smoking cessation intervention either directly or indirectly (e.g., via the mother).

We excluded studies targeting:

- Users of tobacco-related substances other than cigarettes i.e., cannabis, paan, snuss, snuff, hookah, bidi, kretek or other non-cigarette tobacco-related products.
- Women who were vaping as this can be a smoking cessation tool.
- Women who are preconceptual (e.g., couples seeking IVF/ICSI treatment) or targeting women/partners during the postnatal period only.
- The pregnant woman only or targeted a partner or family member where the index pregnant woman is a non-smoker.

In an adaptation to our registered protocol [19] we also excluded studies not written in English.

#### Types of intervention

Interventions were expected to include but not be limited to those incorporating smoking cessation information, counselling or behavioral support, financial incentives, vaping/e-cigarettes, and nicotine replacement therapies (NRT).

#### Control/comparison groups

We accepted any control group/condition with which the intervention was compared. This was expected to include at least one of:

- Intervention targeting pregnant woman only.
- Usual care for smoking cessation in pregnancy.
- Control timepoint e.g., data collected from target population prior to implementation of the intervention.

#### Outcomes

Studies were required to report maternal smoking cessation during pregnancy, assessed either with an objective or subjective measure of smoking cessation, expected to include but not be limited to:

1. Objective measures: e.g., exhaled carbon monoxide, urinary/serum cotinine.
2. Subjective measures: self-reported quitting e.g. number of days not smoking, number of participants who reported smoking in last 7 days (dichotomous: yes/no)

Studies could also include an objective or subjective assessment of partner smoking cessation. We excluded those studies where the only measurement of smoking cessation was assessed in the postnatal period.

#### Study design

We included studies with a control group/timepoint. This was expected to include randomised controlled trials (RCTs), controlled non-randomised studies, controlled cohort studies and before-and-after studies. We took an inclusive approach and included feasibility and pilot studies if effects of the intervention were reported. We excluded studies without a control group/control timepoint. Qualitative studies were not included in this review.

### Information sources and search strategy

Search terms relating to the PICOS criteria were developed with assistance from a specialist medical subject librarian. These included synonyms such as, but not limited to, pregnant, smoking cessation, nicotine replacement, inhalator, lozenge, vaping, incentivisation, and couples (see Additional file 1 for Medline example strategy). The following MESH terms were included: pregnancy, smoking cessation or tobacco use cessation devices, nicotine replacement, and couple-based/family/partner. The search was devised in Medline, with thesaurus terms adapted for other databases. Searches were conducted with no language restrictions and no limit on study design from database inception to 14th May 2025. The search was restricted to humans and adults and applied to 6 electronic databases and a trial registry: MEDLINE, Embase, Emcare, AMED, BNI, CINAHL, PsycINFO, Cochrane Register of Controlled Trials.

### Study selection

We downloaded citations and screened them using Covidence [21]. Two authors screened all titles and abstracts, and full texts of retained citations using the inclusion and exclusion criteria. During the full-text screening stage, excluded studies were allocated to an exclusion reason by two authors using the first criterion met. Reference lists of relevant systematic reviews identified at full text stage were reviewed by hand and are identified as additional sources in the PRISMA diagram. Disagreements were resolved through discussion with the senior authors (AD, CB). Where citations were abstracts for conferences, trial registrations or protocols, the reviewers made efforts to identify whether the study had been published in a peer-reviewed publication by contacting relevant authors on two occasions.

### Data extraction

A standardised, piloted data extraction form was used to extract data from the included studies for the assessment of study quality and evidence synthesis. Two researchers undertook all extraction and discrepancies were resolved through discussion. The extracted information included basic publication details (author and date of publication); study setting (country, country income status; study population and participant demographics (age, parity, education, ethnicity where stated); baseline characteristics (cigarettes smoked per day, relationship of pregnant woman to other participant/s (i.e. partner/family/household member)); nature of intervention; intervention target (woman, partner, woman and partner, direct or indirect targeting); study design; number of participants included in analysis; outcomes; outcome measurement tools and timepoints of measurement. Following initial review of the intervention types, two researchers categorised them thematically into information, behavioural support, NRT and ‘other’.

### Risk of bias

Risk of bias for RCTs and cohorts with a control group was assessed using the Cochrane EPOC risk of bias tools for studies with a control group, and the tool for interrupted time series designs for before and after studies [22].

### Adequacy of intervention description

The TIDieR checklist was used to extract data on adequacy of intervention description [23]. It examines in detail the replicability of the intervention by determining whether precise details of the intervention are provided. Extracted data were: intervention name; theory and evidence base for the intervention; what was the intervention; procedures used; intervention provider and skills/training received; how, where, when and frequency of delivery; tailoring reported; reported modifications and fidelity of delivery.

### Data synthesis

All reported measures of effect (Odds Ratio, Risk Ratio and Risk Difference) as well as counts/% of events were extracted. It was intended for studies to be analysed by intervention type and outcome. If we identified at least two studies with combinable results, a meta-analysis would be undertaken in STATA or RevMan. A fixed effect or random effect model would be selected based on the level of heterogeneity between studies. However, due to clinical and methodological heterogeneity identified within the dataset it was not possible to combine data and undertake meta-analyses. Therefore, we summarised the data using a narrative synthesis approach [24].

## Results

### Study selection

**Fig. 1:**
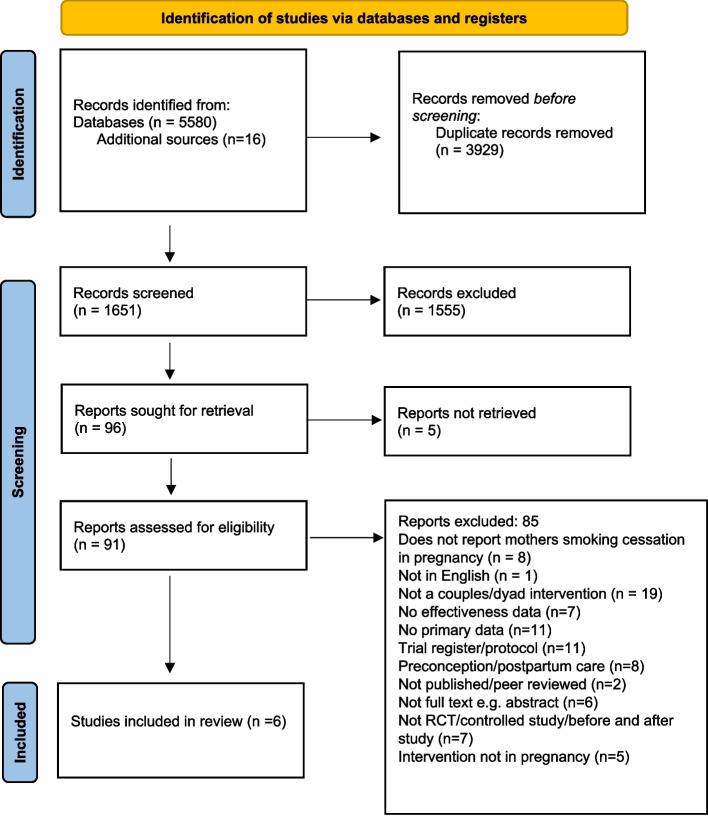
PRISMA Flowchart

The PRISMA flow diagram is provided in Fig. 1. Following de-duplication, 1,651 titles and abstracts were screened, with 91 full texts reviewed. Six studies met the inclusion criteria.

There was methodological and clinical heterogeneity across studies relating to interventions, extent to which the partner/family member was directly or indirectly targeted, outcome assessment timepoints and outcomes assessed. Therefore, it was not possible to perform meta-analysis. A narrative summary is used to describe the characteristics and findings of each included study.

### Characteristics of included studies

Of the six included studies, four were randomised controlled trials [25–28] and one was a non-randomised comparative study [29]. One study was a before and after study [30].

Five studies were undertaken in high income countries [25–29] and one study was undertaken in a middle-income country [30]. All studies targeted the pregnant woman and her partner, one study also included another household or family member (relationship not specified by authors) [30].

There was variation in participant demographic characteristics across studies (see Table 2). One study specifically targeted Aboriginal and Torres Strait Islanders [26], however in all other studies participants were predominantly of White ethnicity, representing the majority population in that country [25, 27–30]. In two studies the majority of mothers were, or the mean age was, under 25 years [25, 29]. There was variation in the educational level of participants with two studies reporting that over 50% of participants had a high school or higher level of education [25, 30].

### Risk of bias of selected studies

**Fig. 2:**
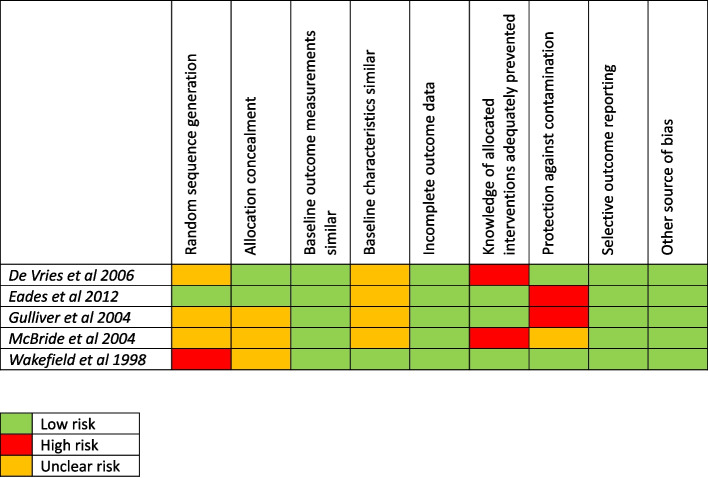
EPOC Risk of Bias assessment for studies with a control group

**Fig. 3:**
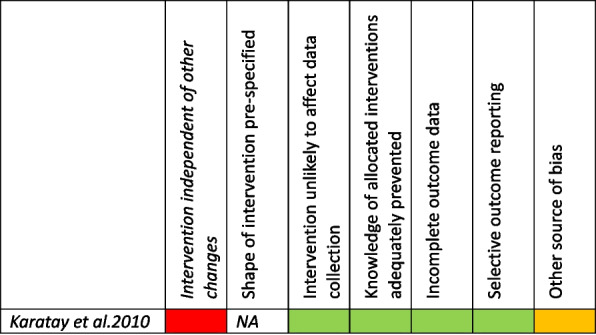
EPOC Risk of Bias assessment for studies without a control group

All studies had at least one domain that was at high risk of bias (Fig. 2, Fig. 3). Random sequence generation, allocation concealment and baseline characteristics data were often unclear. Baseline outcome measurements were all classified as low risk due to the inability to measure smoking cessation at baseline.

### Quality of intervention reporting

**Fig. 4:**
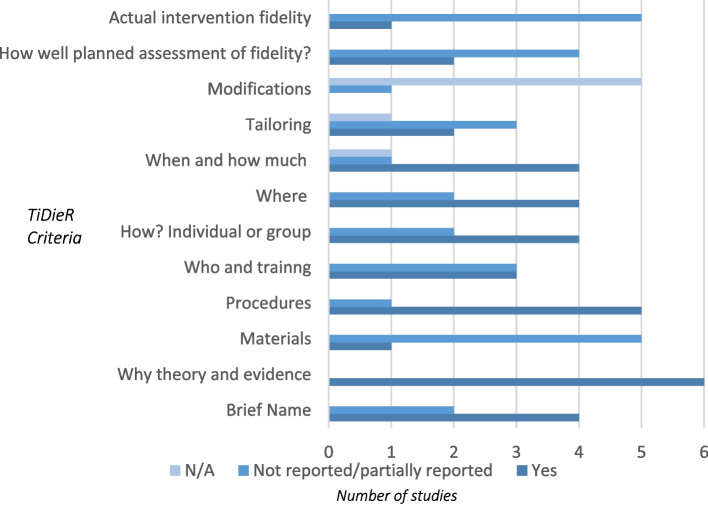
Quality of intervention reporting based on the TIDieR Criteria

Overall, studies provided detailed descriptions of intervention content in relation to some domains of the TIDieR criteria including the theory and evidence based used, procedures, who was targeted and frequency with which the intervention was delivered (Fig. 4). Key limitations to descriptions that could limit replication of them included: descriptions of materials, how interventions were tailored to participants and limited attempt to achieve or report fidelity of delivery (Fig. 4).

### Interventions

The components of interventions are summarised in Table 1. Only one study delivered the same intervention to the woman and her partner [28], therefore interventions are described in relation to each target group.

**Table 1:** Components of interventions

	**Mother intervention**	**Partner intervention**	**Approach to partner**
de Vries et al. 2006 [27]	Face-to-face behavioural support Written/self-help materials	Written materials	Via woman
Eades et al. 2012 [26]	Face-to-face behavioural support NRT	Letter inviting to attend next appointment to support Written/self-help materials	Via woman
McBride et al. 2004 [25]	Telephone behavioural support Written/self-help materials	Telephone behavioural support Written/self-help materials NRT	Directly
Karatay et al. 2010 [30]	Face-to-face behavioural support	Written materials, attempt to meet Face-to-face to discuss passive smoking	Directly
Gulliver et al. 2004 [28]	Face-to-face behavioural support as couple Childcare and ‘cab’ vouchers Incentives Written/self-help materials	Face-to-face behavioural support as couple Written/self-help materials	Directly
Wakefield et al. 1998 [29]	Demonstration of smoking on fetal heart Behavioural support Written/self-help materials	Written/self-help materials	Via woman

### Interventions targeting pregnant women

All studies described using behavioural support in the form of face-to-face or telephone counselling. Four studies provided additional self-help materials in the form of information booklets/leaflet [25, 27–29]. One study provided nicotine replacement therapy (NRT) for women who were still smoking after behavioural support [26]. One study trialled a demonstration of the effect of smoking on the fetal heart alongside behavioural support [29]. One study provided women with incentives in the form of raffle tickets to win a car seat for attendance, as well as childcare and taxi vouchers [28].

### Interventions targeting partners

Three studies approached partners indirectly via the woman (for example, asking the woman to give her partner a leaflet) [26, 27, 29] and three studies approached partners directly (for example, involving them in face-to-face behavioural support) [25, 28, 30].

Written materials and behavioural support were predominant components of interventions offered for partner. All studies provided partners with a form of written or self-help materials, including booklets, leaflets and companion videos [25–30]. Four studies either provided or invited partners to receive face to face or telephone behavioural support to quit smoking [25, 26, 28, 30]. Behavioural support consisted of: functional analysis to identify individualised behaviour change plans with a clinical psychologist [28], motivational interviewing [30], and educational explanations of the harm of active and passive smoking [29]. One study offered nicotine replacement patches alongside behavioural support [25].

### Control groups

Only one study directly compared targeting the woman alone versus targeting her with her partner [25]. Four studies delivered the intervention to the couple and compared it with routine care [26–29]. One study had no control group (before and after study) [30].

### Outcome measures and measurement timepoints for mothers

All six studies included an objective measure of smoking cessation for the mother [25–30]. Objective measures and cut-offs used to define smoking cessation varied: four studies used urinary cotinine but with differing cut-offs used to define cessation, of < 100 ng/mL [29], < 250 ng/mL [26, 27] and < 500 ng/mL [30]. In one study, urinary cotinine was assessed in 30% of the sample to validate self-reported smoking behaviour, although biochemical validation results were unusable [27]. Two studies measured CO from expired air with differing cut-offs used to define cessation as < 9 ppm and < 10 ppm [28, 30]. One study used salivary metabolite to measure cessation but did not specify the metabolite tested or cut-off values to indicate cessation [25].

All six studies used a subjective measure alongside objective measures [25–30]. Tools to measure cessation varied and included any cigarettes smoked in last 7 days [25–27, 29], and quit for 24 h since last measurement contact [27].

#### Measurement timepoints

Three studies based measurements on gestation, which ranged from 28 to 36 weeks [25, 26, 29].Three studies measured cessation with reference to time post intervention, potentially resulting in varied gestations at which measurements were taken within the study [27, 28, 30].

### Outcome measures for partners

Two studies did not report partners’ smoking status at baseline or quit attempt [26, 28], however of these one reported ‘exposure to environmental smoke’ which could be a proxy for partner/household smoking and is included below [30]. One study collected baseline data on partners’ smoking status but did not collect follow-up data to assess cessation [26]. One study asked women to report their partners’ smoking [27]. One study measured partner cessation using self-report at 24–26 weeks and again at 32–34 weeks’ gestation but did not biochemically validate an attempt with urinary cotinine as was the procedure for the women [29]. One study assessed both subjective and objective cessation, self-reported quitting and attempts and biochemical validation with a saliva sample [25].

### Effectiveness

A summary of the findings of each study is presented in Table 2.

**Table 2:** Study design and findings

Study	Study Design	Participant characteristics	Intervention (I)	Control group/s (C)	Maternal cessation measure, cessation threshold and & timepoint	Cessation outcomes women	Cessation outcomes partner
McBride et al. [25] USA	RCT **(*n* = 583)**	**Education:** More than high school: 52% **Ethnicity:** 77% White **Age (mean):** 24	**Women:** (*n* = 193) 3 behavioural support sessions based on motivational interviewing in pregnancy delivered by health advisor. Late relapse prevention kit (booklet and gift item) **Partners:** Encouraged to support woman/quit themselves, NRT	Usual care (C1) (*n* = 198); Non-partner assisted intervention (Woman only (C2)) (*n* = 192)	**Subjective/Objective:** Self-report cessation in past 7 days, validated by salivary metabolite (unknown metabolite) **Timepoint:** 28 weeks gestation	**C1:** 61%; **C2:** 56%; **I:** 58%, *p* = 0.77	**C1:** 5%, **C2**: 9%, **I**: 15%; *p* = 0.02
De Vries et al. [27] The Netherlands	RCT **(*n* = 318)**	**Education:** Low education (not defined): **I:** 47.1%, **C:** 61.6%; High education: **I:** 14.7%, **C:** 14.6% **Ethnicity:** Not reported **Age (mean): I:** 28.6 years, **C:** 28.4 years	**Women: (***n* = 141) Face to face behavioural support × 2 contacts, based on 7 step Health Counseling (Minimal intervention strategy) MIS protocol video, self-help materials, relapse prevention **Partners:** Booklet offered via woman	Usual care (*n* = 177)	**Subjective:** Self-report cessation in past 7 days, quit attempt of at least 24 h since last measurement contact **Objective:** Urinary Cotinine < 250 ng/mL (biochemical validation undertaken on 30% of participants). **Biochemical validation unusable** **Timepoint:** (Variable) 6 weeks after intervention	**7 day abstinence C:** 7%; **I:** 19%; (OR = 6.67, 95% CI: 1.81–24.59 *p* = 0.004)	72% of women reported partner to be a smoker at timepoint. Intervention no effect
Eades et al. [26] Australia	RCT **(*n* = 263)**	**Education:** Not reported **Ethnicity:** All participants Aboriginal and Torres Strait Islanders **Age (mean):** Not reported	**Women:** (*n* = 148) Scripted invitation from doctor, advised to go cold-turkey at initial antenatal appointment, up to two further follow-up appointments + fridge magnet reminder. NRT offered if still smoking 7–10 days post intervention. **Partners:** Letter, invited to attend next appointment	Usual care (*n* = 115)	**Subjective/Objective:** Self-report cessation in past 7 days validated by Urinary Cotinine < 250 ng/mL **Timepoint:** 36 weeks gestation	**Per protocol: C:** 95%; **I:** 89%; (RR = 0.93, 95%CI: 0.86–1.08, *p* = 0.21) **Intention to treat: C**: 97%, **I**:93% (RR = 0.95, 95%CI: 0.90–1.01, *p* = 0.21)	Not reported
Gulliver et al. [28] USA	Pilot RCT **(*n* = 20)**	**Education:** 10–16 years (mean 12.9) **Ethnicity:** 75% White, 13% Hispanic, 6% Black **Age (mean):** 26.7 years	**Women + Partner:** Group counselling with partner (based on Community Reinforcement Approach) + raffle tickets, childcare and taxi vouchers for attendance + baby equipment coupon if quit. Multiple contacts, intensity not defined	**Women:** Group counselling without partner + raffle tickets, childcare and taxi vouchers for attendance + baby equipment coupon if quit	**Subjective/Objective:** Quit by quit date; self-report abstinent 1 month later validated by expired CO < 10 ppm **Timepoint:** quit date; 1 month post-quit	**I:** Quit by quit date (χ^2^(1, *n* = 20) = 4.4, *p* < 0.05), 1 month abstinence: (χ2(1), *n* = 20) = 4.4, *p* < 0.05)	Not reported
Wakefield et al. [29] Australia	Non-randomised comparative	**Education:** Not reported **Ethnicity:** Not reported **Age (mean): C1:** 24.5 years**; C2:** 25.05 years**, I:** 24.9 years	**Women**: (*n* = 110) Demonstration of impact of smoking on fetal heart – 1 × contact.) **Partner:** Written information provided via woman	Usual Care (C1) (*n* = 103)—historical control group in same antenatal clinic Usual care (C2) (*n* = 110)—in second antenatal clinic usual care	**Subjective/Objective:** Self-report sustained cessation from 24–26 weeks to 32–24 weeks; Point prevalent abstinence 32–34 weeks validated by Urinary cotinine < 100 ng/mL **Timepoint:** 32–24 weeks gestation	Sustained cessation: **C**: 2.8%; **I:** 9.3% (aOR: 1.7, 95% CI 0.97–2.96; *p* =.11) Point-prevalent abstinence:** C**: 5.1%; **I:** 10.1%, (aOR: 1.4, 95% CI 0.92–2.23; *p* =.11)	Point-prevalent Abstinence: **I:** 1.8%; **C:** 2.1%; (aOR = 0.87, 95% CI = 0.05–14.4, p not reported)
Karatay et al. [30] Turkey	Before and after study **(*n* = 38)**	**Education:** high school 34.2%; University: 18.4% **Ethnicity:** Not reported **Age**: 47.4% 20–25 years	**Women:** Behavioural support with motivational interviewing in 8 home visits **Partners/family/household member**: brochure explaining harms and meetings with others in home who smoked	No control group	**Subjective:** Self-reported quit **Objective:** Urinary cotinine < 500 ng/mL, Expired CO < 9 ppm **Timepoint: (variable)** 3 months post intervention	3 months, 39.5% of women had quit, authors report that this is significantly different from the expected value (c^2^(1) = 17.85, *p* <.001)	Environmental smoke exposure: enrolment (86.8%) to intervention end (44.7%, *p* <.05)

### RCTs

Of the four included RCTs, two reported that more women in the intervention arm had quit smoking compared with those in the control group [27, 28] and two described no effect of the intervention compared with the control group/s on women’s antenatal smoking cessation [25, 26].

In the only study directly comparing partner-supported versus woman only smoking cessation intervention on cessation, McBride et al. [25] undertook a three-arm RCT comparing 1) usual care (*n* = 198), 2) woman only (*n* = 192; 6 behavioural support sessions based on motivational interviewing) and 3) partner assisted arm (*n* = 193) wherein partners were encouraged to support the woman’s quit attempt/quit themselves where appropriate. Self-reported smoking status for women and partners was measured and verified with saliva samples at 28 weeks gestation (metabolite not reported) where they had reported not smoking in the previous 7 days. Intention-to-treat analyses showed no differences by condition in women’s reports of abstinence at any follow-up. They report increased cessation in the intervention versus control group for partners.

De Vries et al. [27] randomised pregnant women from midwifery practices across provincial networks to clustered pairs to receive either 1) Intervention: face-to-face behavioural (counselling) support, a video and self-help written materials and relapse prevention booklet for the woman and a booklet offered to partners via the woman (*n* = 318); 2) Control: usual care (*n* = 141). The authors report that point-prevalent abstinence (smoking in past 7 days) was greater in the intervention versus control arm at 6 weeks post intervention. At intake and both post-tests, approximately 72% of women reported their partner to be a smoker with intervention reported to have no effect on smoking cessation.

Eades et al. [26] randomised pregnant women to: 1) Intervention (*n* = 263): scripted invitation from doctor to quit smoking, advised to go ‘cold turkey’ and given a follow-up appointment reminder card and fridge magnet. They received a letter for other smoking household members and were told to bring a support person/partner to next appointment. For women who were still smoking at 7–10 days post invitation, NRT was discussed and/or commenced; 2) Control group participants received usual care (UC; *n* = 115). The authors report no difference between groups for cotinine-validated self-report of not smoking in last 7 days at 36 weeks gestation. Intention to treat analysis indicated higher smoking rates in the control group compared with the intervention. Partner/household member cessation was not reported.

In a randomised controlled pilot study Gulliver et al. [28] randomised women and their partners to 1) group counselling with their partner, 2) group counselling without partner. Group counselling was a 60-min manual guided behavioural support session conducted by a clinical psychologist. Participants were given a raffle ticket for a car seat, childcare and taxi vouchers for attendance (regardless of smoking status). Women demonstrating abstinence were given a coupon for varied baby equipment that could be accumulated for larger incentives. In the intervention arm partners were given a tip sheet describing partner support and effective communication. Abstinence was measured as expired CO. The authors report that intervention group women were more likely to quit on their scheduled quit date and to remain abstinent one month later compared with control group participants. Partner cessation was not reported.

### Non-randomised studies

Wakefield and colleagues [29] compared: 1) demonstration of the impact of smoking on the fetal heart to the pregnant woman and written materials for the partner provided via the woman (*n* = 110), with 2) a historical usual care control group from the same antenatal clinic (*n* = 110). Data were collected from a second antenatal clinic at both time points (*n* = 103 and *n* = 110) to control for time-related effects. The authors report no time effect. They compared sustained abstinence between 32 and 34 weeks gestation and point-prevalent abstinence (24–26 weeks and 32–34 weeks gestation) between intervention and control participants using urinary cotinine. Women’s sustained cessation between 32–34 weeks gestation was greater in the intervention group compared with controls. Point prevalent abstinence at 32–34 weeks was higher in the intervention versus control group. Point prevalence quit rates for intervention and control group partners did not differ in late pregnancy.

In a before and after study Karatay et al. [30] provided face-to-face behavioural support in the form of motivational interviewing in eight home visits to women before 16 weeks gestation (*n* = 45). Inclusion of partners was via a brochure explaining harms of smoking and arranging face-to-face meetings with the others at home who smoked. Self-report quit attempts were verified with expired CO and urinary cotinine at 3 months post initial intervention. At 3 months, 39.5% of the women had quit and the authors report that this is significantly different from the expected value. A decrease in women’s smoking (number of cigarettes) was also reported. The authors report a reduction in environmental smoke exposure of the women from enrolment (86.8%) to end of intervention (44.7%; *p* < 0.05).

## Discussion

We have identified only six studies evaluating couple/household-targeted interventions to support smoking cessation in pregnant women where both parties smoke. Two RCTs [27, 28], one non-randomised comparative study [29] and one before and after study [30] report an effect of the tested intervention on smoking cessation in the pregnant woman. Interventions delivered to women and reported by the authors to be associated with increased likelihood of antenatal smoking cessation compared with usual care were: face-to-face behavioural support, a video, self-help written materials [27], a 60-min manual-guided behavioural support session conducted by a clinical psychologist with their partner[28], demonstration of the impact of smoking on the fetal heart[29], and face-to-face motivational interviewing within at-home visits to women [30]. Interventions targeting partners and reported by the authors to be associated with increased likelihood of women’s antenatal cessation were: written self-help materials [27, 29, 30] and attendance at behavioural support session with a clinical psychologist[28]. However, of these studies two reported no effect of written materials on partner smoking [27, 29] and one did not measure the partner’s smoking status [28]. One study reported a reduction in environmental smoke exposure of the women from enrolment to end of intervention [30].

This systematic review demonstrates that a limited number of studies have been undertaken targeting couples and households and only one recent study has targeted couple cessation, despite the important predictive role of partners’ smoking in pregnant women’s cessation and relapse [8, 11, 31]. In comparison, recent systematic reviews have identified considerably more (*n* = 102) studies delivering psychosocial interventions for pregnant women who smoke [13], but a similarly small number of studies (*n* = 5) targeting the smoking partners of non-smoking pregnant women [32]. Notably, our findings indicate that there have been no recent efforts to target couple/household smoking in pregnancy, with the most recent study undertaken in 2012. The paucity of evidence around partner smoking interventions during pregnancy may reflect a lack of recognition of the importance of partner cessation for achieving maternal cessation and achieving a smoke-free environment. It may also reflect practical barriers to engaging partners and wider family members in smoking cessation interventions and research during pregnancy, when many pregnancy healthcare contacts are with the mother alone. Finally, it is possible that interventions are being delivered to couples and families, but are not evaluated for publication in the scientific literature.

Within this review, only one study directly compared targeting the woman and her partner versus the woman only [25], with all remaining studies comparing couple-targeted interventions with usual care. Without targeting couples, and without comparing the impact of targeting couples/households with targeting women only, we cannot identify whether this is an effective means through which to change pregnant women’s smoking behaviour. One potential reason for this lack of direct comparison is that partners are rarely directly targeted within the intervention, with half of the included studies targeting partners indirectly through the pregnant woman [26, 27, 29]. Partners were also offered differing interventions from the pregnant woman in five studies [25–27, 29, 30], which in some cases involved only written materials [27, 29]. This may mean that partners do not even receive the intended intervention. This potential for lack of intervention receipt [33] could limit intervention effectiveness for partners and poses challenges for uptake of interventions in clinical practice. Indeed, no studies reported whether partners engaged with the intervention, and only three studies assessed intervention impact on partner cessation [25, 26, 29]. This is a missed opportunity to evaluate whether partner intervention exposure and cessation mediates intervention effects on the pregnant woman’s cessation and relapse, and therefore whether it is beneficial to women’s cessation to expend resource on supporting partner cessation. Together, these findings highlight a need to directly target partners within interventions and test them within high-quality RCTs in which couple/household interventions are compared with current usual care (typically woman only). Additionally, assessing both the woman’s and partner’s cessation will provide understanding as to whether targeting couples/households is effective for achieving smoking cessation for pregnant women. Innovative methods for engaging partners during pregnancy healthcare contacts are needed.

There was variation in the demographic characteristics of the study participants, with only one study specifically targeting women from a minoritised ethnicity group (Aboriginal and Torres Strait Islanders) [26]. The majority of participants were from White ethnic/racial backgrounds and most studies indicated that participants were typically younger mothers aged under 25 or 30 years. This reflects patterns of smoking during pregnancy in the UK population and other high-income countries where the population is predominantly Caucasian [4, 34], but suggests a need to investigate the effectiveness of these interventions and to ensure they are appropriate in other countries or for use with other ethnic groups.

A further methodological issue identified within this review is considerable heterogeneity in the tools used to assess, and definitions of smoking cessation applied. A range of subjective and objective measures were used across studies, with differing cut-off values applied to urinary cotinine and exhaled CO. This lack of standardisation of outcome assessment prevented comparison between studies [35]. In a recent Delphi survey, experts agreed that preferred biochemical validation methods were carbon monoxide (expired air) and cotinine (saliva), however, no threshold values for cessation were discussed or defined [36]. This suggests that there will be continued heterogeneity in how cessation is evaluated, and that challenges with synthesising evidence to compare intervention effectiveness will persist.

Within the current review we have identified no use of up-to-date interventions such as financial incentives or e-cigarettes offered to women and their partners. This is likely to be due, in part, to the lack of recent studies identified. However, it may also result from uncertainty about the safety of e-cigarettes in pregnancy [14]. Notably, one recent study of NRT and e-cigarette users during pregnancy suggests no increased incidence of perinatal death, preterm labour or low birth weight with e-cigarette use and a comparable safety profile to NRT [37]. Furthermore, no studies in this review offered direct financial incentives for attendance or in response to a successful quit attempt. There is evidence to support the use of both e-cigarettes and financial incentives in the general population, and they are increasingly being used to support smoking cessation both in the general population and in pregnant women [7, 38, 39]. The independent UK government report ‘Making smoking Obsolete’ [40] recommends dedicated funding for financial incentives for all pregnant women, and within local Tobacco Dependency Treatment Services, there is increasing use of financial incentives and e-cigarettes to support pregnant women to stop smoking. These are often provided as pilot schemes alongside behavioural support and combination NRT [41]. Since the NHS long-term plan specifically calls for new treatment pathways for pregnant women *and* their partner, the utility of e-cigarettes and incentives to aid smoking cessation for couples during pregnancy should be explored [42].

### Strengths and limitations

We used a systematic, valid, and reliable process to search, select studies for inclusion and extract data, which is likely to have resulted in identification of all relevant publications in this area. We took an inclusive approach, incorporating both randomised controlled trials and observational studies, to provide a comprehensive understanding of the current evidence base, and to enable us to highlight potential areas for improvement in the development of effective couple/household targeted interventions for smoking cessation in pregnancy. We also used established tools to systematically evaluate risk of bias and to extract data on the quality of intervention reporting.

Limitations of the methodology of the current review include that we did not explore the grey literature. Interventions to support women and their partners may be described in local reports or used in practice without their effectiveness being reported. Smoking cessation services may be able to provide insight about how this is being addressed in antenatal care. There is also potential for publication bias [43], which prevents the identification of ineffective interventions. We did not include qualitative studies; these could signal potentially effective interventions to be explored in future RCTs. Finally, we did not explore the effectiveness of interventions for partners’/household members’ cessation where the woman was a non-smoker, and did not explore the effects of interventions on sustained cessation beyond the end of pregnancy. The impacts of passive smoking on maternal and infant outcomes are well-documented [44, 45], and there is evidence that up to 43% of women who quit smoking in pregnancy relapse after birth [46]. Further systematic reviews should be conducted to update knowledge of the most effective interventions to address these issues.

### Implications for policy, research and practice

This review demonstrates that there is a limited evidence base to inform the clinical use of interventions to support couples/households to quit smoking in pregnancy. The documented impact of partner smoking on pregnant women’s ability to quit smoking during pregnancy means it is vital that policymakers consider delivery and evaluation of interventions to support this, and that clinical staff consider the importance of household cessation where a pregnant woman smokes, so that appropriate cessation support is recommended. High-quality, robust RCTs with adequate sample sizes and appropriate control groups are needed to establish efficacy of couple/household targeted interventions, and cost-effectiveness of the intervention to inform commissioning decisions. These interventions should be based on the best available current interventions used within and outside pregnancy, where considered safe, for example behavioural support, financial incentives and potentially e-cigarettes.

## Conclusions

There has been very limited exploration of couple/household-targeted interventions to support women to quit smoking in pregnancy where partners and family members are also smokers. Studies were at risk of bias and interventions are heterogenous, poorly described, with effectiveness assessed using differing measures with differing cessation thresholds, preventing meta-analysis. Interventions for which the authors report an association with reduced smoking in pregnant women include behavioural support [27], including behavioural support for couples conducted by a clinical psychologist [28], demonstration of the effect of smoking on the fetal heart [29] and written self-help materials for partners[27, 29, 30]. These could be explored in future high quality RCTs. The lack of recent studies in this area highlights the need to establish the evidence base for the use of up-to-date cessation support tools and techniques for couples in pregnancy. Consistent measurement of cessation for all intervention recipients and expert-agreed objective cessation assessments are needed to ensure robustness of findings and support future evidence synthesis.

## Supplementary Information

Additional file 1.

Additional file 2.

## Data Availability

The datasets used and/or analysed during the current study are available from the corresponding author on reasonable request.

## References

[1] Mitchell EA, Milerad J. Smoking and the sudden infant death syndrome. Rev Environ Health. 2006;21(2):81–103. 10.1515/reveh.2006.21.2.81. 16898673 10.1515/reveh.2006.21.2.81

[2] Lyus L. Impact of smoking on future rates of stillbirth, neonatal and infant mortality and poor birth outcomes in England 2018. Available from: https://ash.org.uk/uploads/Smoking-in-pregnancy-adverse-outcomes-paper-June-2018.pdf ? Accessed: 17/12/2024.

[3] Sun J, Liu X, Zhao M, Magnussen CG, Xi B. Dose-response association between maternal smoking during pregnancy and the risk of infant death: a nationwide, population-based, retrospective cohort study. EClinicalMedicine. 2023;57:101858. 10.1016/j.eclinm.2023.101858. 36879656 10.1016/j.eclinm.2023.101858 PMC9984774

[4] Lange S, Probst C, Rehm J, Popova S. National, regional, and global prevalence of smoking during pregnancy in the general population: a systematic review and meta-analysis. Lancet Glob Health. 2018;6(7):e769–76. 10.1016/S2214-109X(18)30223-7. 29859815 10.1016/S2214-109X(18)30223-7

[5] NHS England. Saving babies lives 2016. Available from: https://www.england.nhs.uk/long-read/saving-babies-lives-version-3/ Accessed: 29/09/2025.

[6] NHS Digital. Statistics on Women's Smoking Status at Time of Delivery: England, Quarter 3, 2024/25 2024. Available from: https://digital.nhs.uk/data-and-information/publications/statistical/statistics-on-women-s-smoking-status-at-time-of-delivery-england/statistics-on-womens-smoking-status-at-time-of-delivery-england-q3-2024-25. Accessed: 10/9/2025.

[7] Notley C, Brown TJ, Bauld L, Hardeman W, Holland R, Naughton F, et al. Development of a complex intervention for the maintenance of postpartum smoking abstinence: process for defining evidence-based intervention. Int J Environ Res Public Health. 2019;16(11). 10.3390/ijerph16111968. 10.3390/ijerph16111968 PMC6603989 31163663

[8] Riaz M, Lewis S, Naughton F, Ussher M. Predictors of smoking cessation during pregnancy: a systematic review and meta-analysis. Addiction. 2018;113(4):610–22. 10.1111/add.14135. 29235189 10.1111/add.14135

[9] Posse CM, Val A, Míguez M. Social support and women’s smoking during the perinatal period: a systematic review. Clin Health J Empir Res Psychol. 2025;26(1):25–35.

[10] Roman-Galvez RM, Amezcua-Prieto C, Olmedo-Requena R, Lewis-Mikhael Saad AM, Martinez-Galiano JM, Bueno-Cavanillas A. Partner smoking influences whether mothers quit smoking during pregnancy: a prospective cohort study. BJOG. 2018;125(7):820–7. 10.1111/1471-0528.14986. 29052334 10.1111/1471-0528.14986

[11] Appleton PL. Partner smoking behaviour change is associated with women's smoking reduction and cessation during pregnancy. Br J Health Psychol 1998 10.1111/J.2044-8287.1998.TB00580.X.

[12] DiClemente CC, Dolan-Mullen P, Windsor RA. The process of pregnancy smoking cessation: implications for interventions. Tob Control. 2000;9(Suppl 3):III16-21. 10.1136/tc.9.suppl_3.iii16. 10982900 10.1136/tc.9.suppl_3.iii16 PMC1766302

[13] Chamberlain C, O’Mara-Eves A, Porter J, Coleman T, Perlen SM, Thomas J, et al. Psychosocial interventions for supporting women to stop smoking in pregnancy. Cochrane Database Syst Rev. 2017;2(2):CD001055. 10.1002/14651858.CD001055.pub5. 28196405 10.1002/14651858.CD001055.pub5 PMC6472671

[14] Coleman T, Chamberlain C, Davey MA, Cooper SE, Leonardi-Bee J. Pharmacological interventions for promoting smoking cessation during pregnancy. Cochrane Database Syst Rev. 2015;12:CD010078. 10.1002/14651858.CD010078.pub2. 10.1002/14651858.CD010078.pub2 26690977

[15] Stanton WR, Lowe JB, Moffatt J, Del Mar CB. Randomised control trial of a smoking cessation intervention directed at men whose partners are pregnant. Prev Med. 2004;38(1):6–9. 10.1016/j.ypmed.2003.09.021. 14672636 10.1016/j.ypmed.2003.09.021

[16] Pollak KI, Denman S, Gordon KC, Lyna P, Rocha P, Brouwer RN, et al. Is pregnancy a teachable moment for smoking cessation among US Latino expectant fathers? A pilot study. Ethn Health. 2010;15(1):47–59. 10.1080/13557850903398293. 20013439 10.1080/13557850903398293 PMC3637962

[17] Faseru B, Richter KP, Scheuermann TS, Park EW. Enhancing partner support to improve smoking cessation. Cochrane Database Syst Rev. 2018;8(8):CD002928. 10.1002/14651858.CD002928.pub4. 30101972 10.1002/14651858.CD002928.pub4 PMC6326744

[18] Hemsing N, Greaves L, O’Leary R, Chan K, Okoli C. Partner support for smoking cessation during pregnancy: a systematic review. Nicotine Tob Res. 2012;14(7):767–76. 10.1093/ntr/ntr278. 22180588 10.1093/ntr/ntr278

[19] Davies A, Burden C, Overton T, Merriel A. Couples-based interventions for smoking cessation in pregnancy - A systematic review. https://www.crd.york.ac.uk/PROSPERO/view/CRD42022196297. PROSPERO. 2024. Available from: https://www.crd.york.ac.uk/PROSPERO/view/CRD42022196297. 10.1186/s12889-026-27484-0 PMC13325784 42104326

[20] McKenzie JE, Brennan SE, Ryan RE, Thomson HJ, Johnston RV, J. T. Chapter 3: Defining the criteria for including studies and how they will be grouped for the synthesis [last updated August 2023]. In: Higgins JPT, Thomas J, Chandler J, Cumpston M, Li T, Page MJ, Welch VA, editors. Cochrane Handbook for Systematic Reviews of Interventions. version 6.5. Cochrane;2024 Available from: https://www.cochrane.org/authors/handbooks-and-manuals/handbook/current/chapter‑03#section‑3‑5 Accessed: 18/9/2025

[21] Veritas Health Innovation. Covidence systematic review software: Melbourne; 2024 Available from: https://www.covidence.org. Accessed: 17/12/2024.

[22] Effective Practice and Organisation of Care (EPOC). EPOC Taxonomy 2015. Available from: https://www.epoc.cochrane.org/epoc-taxonomy Accessed: 02/10/2025

[23] Hoffmann TC, Glasziou PP, Boutron I, Milne R, Perera R, Moher D, et al. Better Reporting of Interventions: Template for Intervention Description and Replication (TIDieR) Checklist and Guide. BMJ. 2016;78(3):e174. 10.1055/s-0037-1600948. 10.1055/s-0037-1600948 29298449

[24] Popay J, Roberts H, Sowden A, Petticrew M, Arai L, Rodgers M, et al. Guidance on the conduct of narrative synthesis in systematic reviews: A product from the ESRC Methods Programme. 2006. Available from: https://www.researchgate.net/publication/233866356_Guidance_on_the_conduct_of_narrative_synthesis_in_systematic_reviews_A_product_from_the_ESRC_Methods_Programme. 10.13140/2.1.1018.4643.

[25] McBride CM, Baucom DH, Peterson BL, Pollak KI, Palmer C, Westman E, et al. Prenatal and postpartum smoking abstinence a partner-assisted approach. Am J Prev Med. 2004;27(3):232–8. 10.1016/j.amepre.2004.06.005. 15450636 10.1016/j.amepre.2004.06.005

[26] Eades SJ, Sanson-Fisher RW, Panaretto K. An intensive smoking intervention for pregnant Aboriginal and Torres Strait Islander women: a randomised controlled trial. Med J Aust. 2013;198(1):23–4. 10.5694/mja12.11298. 23330759 10.5694/mja12.11298

[27] de Vries H, Bakker M, Mullen PD, van Breukelen G. The effects of smoking cessation counseling by midwives on Dutch pregnant women and their partners. Patient Educ Couns. 2006;63(1–2):177–87. 10.1016/j.pec.2005.10.002. 16406475 10.1016/j.pec.2005.10.002

[28] Gulliver SB, Colby SM, Hayes K, Raffa SD. Tobacco cessation treatment for pregnant smokers: incorporating partners and incentives. Med Health R I. 2004;87(1):9–12. 14989074

[29] Wakefield M, Jones W. Effects of a smoking cessation program for pregnant women and their partners attending a public hospital antenatal clinic. Aust N Z J Public Health. 1998;22(3 Suppl):313–20. 10.1111/j.1467-842x.1998.tb01383.x. 9629815 10.1111/j.1467-842x.1998.tb01383.x

[30] Karatay G, Kublay G, Emiroglu ON. Effect of motivational interviewing on smoking cessation in pregnant women. J Adv Nurs. 2010;66(6):1328–37. 10.1111/j.1365-2648.2010.05267.x. 20384640 10.1111/j.1365-2648.2010.05267.x

[31] Jane M, Nebot M, Badi M, Berjano B, Munoz M, Rodriguez MC, et al. Determinant factors of smoking cessation during pregnancy. Med Clin (Barc). 2000;114(4):132–5. 10.1016/s0025-7753(00)71218-8. 10734622 10.1016/s0025-7753(00)71218-8

[32] Duckworth AL, Chertok IR. Review of perinatal partner-focused smoking cessation interventions. MCN Am J Matern Child Nurs. 2012;37(3):174–81. 10.1097/NMC.0b013e31824921b4. 22549421 10.1097/NMC.0b013e31824921b4

[33] Borrelli B, Sepinwall D, Ernst D, Bellg AJ, Czajkowski S, Breger R, et al. A new tool to assess treatment fidelity and evaluation of treatment fidelity across 10 years of health behavior research. J Consult Clin Psychol. 2005;73(5):852–60. 10.1037/0022-006X.73.5.852. 16287385 10.1037/0022-006X.73.5.852

[34] Public Health England. Characteristics of women who stop smoking in pregnancy: experimental analysis of data from the Maternity Services Data Set (MSDS) April 2018 to March 2019. 2019 Available from: https://assets.publishing.service.gov.uk/media/614c3ee2e90e077a2ba1f350/Analysis_characteristics_of_women_who_stop_smoking_in_pregnancy.pdf. Accessed: 18/9/2025.

[35] Behrakis P. Tobacco cessation guidelines for high-risk populations 2017. Available from: https://www.tobaccopreventioncessation.com/pdf-62428-4344?filename=TOB_G_%20Tobacco%20Cessation.pdf Accessed: 18/9/2025.

[36] Cheung KL, de Ruijter D, Hiligsmann M, Elfeddali I, Hoving C, Evers S, et al. Exploring consensus on how to measure smoking cessation. A Delphi study. BMC Public Health. 2017;17(1):890. 10.1186/s12889-017-4902-7. 29162043 10.1186/s12889-017-4902-7 PMC5696733

[37] Pesola F, Smith KM, Phillips-Waller A, Przulj D, Griffiths C, Walton R, et al. Safety of e-cigarettes and nicotine patches as stop-smoking aids in pregnancy: secondary analysis of the Pregnancy Trial of E-cigarettes and Patches (PREP) randomized controlled trial. Addiction. 2024;119(5):875–84. 10.1111/add.16422. 38229538 10.1111/add.16422

[38] Hajek P, Przulj D, Pesola F, Griffiths C, Walton R, McRobbie H, et al. Author correction: electronic cigarettes versus nicotine patches for smoking cessation in pregnancy: a randomized controlled trial. Nat Med. 2023;29(11):2957. 10.1038/s41591-022-02099-1. 36344702 10.1038/s41591-022-02099-1 PMC10667089

[39] Lindson N, Butler AR, McRobbie H, Bullen C, Hajek P, Wu AD, et al. Electronic cigarettes for smoking cessation. Cochrane Database Syst Rev. 2025;1(1):CD010216. 10.1002/14651858.CD010216.pub9. 39878158 10.1002/14651858.CD010216.pub9 PMC11776059

[40] Office for health improvement and disparities. Making smoking obselete 2022. Available from: https://www.gov.uk/government/publications/the-khan-review-making-smoking-obsolete/making-smoking-obsolete-summary#critical-recommendations. Accessed: 18/9/2025.

[41] NHS England. Smoke-free pregnancy incentive scheme. Available from: https://www.england.nhs.uk/ourwork/prevention/tobacco-dependency-programme/national-smoke-free-pregnancy-incentive-scheme/. Accessed: 12/12/2024.

[42] NHS. NHS Long term plan 2024. Available from: https://www.longtermplan.nhs.uk/online-version/overview-and-summary/. Accessed: 18/9/2025.

[43] DeVito NJ, B G. Catalogue of bias: publication bias. BMJ Evid-Based Med. 2019;24:53–4. 10.1136/bmjebm-2018-111107. 30523135 10.1136/bmjebm-2018-111107

[44] Delcroix MH, Delcroix-Gomez C, Marquet P, Gauthier T, Thomas D, Aubard Y. Active or passive maternal smoking increases the risk of low birth weight or preterm delivery: benefits of cessation and tobacco control policies. Tob Induc Dis. 2023;21:72. 10.18332/tid/156854. 37256119 10.18332/tid/156854 PMC10226447

[45] Tarasi B, Cornuz J, Clair C, Baud D. Cigarette smoking during pregnancy and adverse perinatal outcomes: a cross-sectional study over 10 years. BMC Public Health. 2022;22(1):2403. 10.1186/s12889-022-14881-4. 36544092 10.1186/s12889-022-14881-4 PMC9773571

[46] Cooper S, Orton S, Leonardi-Bee J, Brotherton E, Vanderbloemen L, Bowker K, et al. Smoking and quit attempts during pregnancy and postpartum: a longitudinal UK cohort. BMJ Open. 2017;7(11):e018746. 10.1136/bmjopen-2017-018746. 29146659 10.1136/bmjopen-2017-018746 PMC5695489

