# Supplementary material for: A systematic review of couple- or household-targeted interventions for smoking cessation in pregnancy
Source: BMC Public Health. 2026 May 8;26:2009. doi: 10.1186/s12889-026-27484-0 (PMC13325784; doi:10.1186/s12889-026-27484-0)
Supplement: Supplementary file 1 — Additional file 1. [file 12889_2026_27484_MOESM1_ESM.docx]

# **Search Strategy Documentation**

**56-014 Searches devised and run by Kirsten Buckley for Sadie Mullin**

**Search summary**

| **Database Name** | **Platform** | **Date Coverage** | **Date of Search** | **# of results** |
| --- | --- | --- | --- | --- |
| 1. Medline | Ovid | 4/12/2023-14/5/2025 | 14/5/2025 | 30 |
| 2. Embase | Ovid | 4/12/2023-14/5/2025 | 14/5/2025 | 99 |
| 3. Emcare | Ovid | 4/12/2023-14/5/2025 | 14/5/2025 | 31 |
| 4. AMED | ProQuest | 4/12/2023-14/5/2025 | 14/5/2025 | 0 |
| 5. CINAHL | EBSCO | 01/12/2023-31/5/2025 | 14/5/2025 | 12 |
| 6. PsycInfo | ProQuest | 5/12/2023-14/5/2025 | 14/5/2025 | 11 |
| 7. BNI | ProQuest | 5/12/2023-14/5/2025 | 14/5/2025 | 3 |
| 8. Cochrane Trials | Cochrane | 2023-2025 | 14/5/2025 | 21 |

**Total Records = 207**

**Search Strategies**

**1. Medline (Ovid)**

1 (pregnan* or obstetric* or matern* or puerpe* or gestation*).ti,ab.

2 (antenatal* or ante-natal* or antepartum or ante-partum or prenatal* or pre-natal*).ti,ab.

3 (gravid* or nulligravid* or primigravid* or multigravid* or primipar* or multipar*).ti,ab.

4 Pregnancy/ or exp Gravidity/ or exp Parity/ or exp Parturition/ or exp "Pregnancy Maintenance"/ or exp "Pregnancy Outcome"/ or exp "Pregnancy, High-Risk"/ or exp "Pregnancy Complications"/

5 or/1-4

6 exp Smoking/

7 smok*.ti,ab.

8 or/6-7

9 5 and 8

10 Smoking Cessation/ or "Tobacco Use Cessation Devices"/

11 (smok* adj cessat*).ti,ab.

12 (nicotine replacement or ((nicotine or tobacco) adj2 (gum* or lozenge* or patch* or spray*)) or (pharmaco* adj2 (nicotine or tobacco or smoking)) or smoking cessation or incentivisation or incentive or voucher* or SMS or "text messag*" or "text-messag*" or "behavio$ support*" or counselling or vape or vaping or e-cigarette* or electronic cigarette* or inhalator* or (quit* adj (smok* or nicotine or tobacco))).ti,ab.

13 or/10-12

14 (couple*-based or couple* or partner* or spous* or family or families or wife* or husband* or girlfriend* or boyfriend* or house* or household*).ti,ab.

15 9 and 13 and 14

16 limit 15 to dt=20231204-20250514

**2. Embase & Emcare (Ovid)**

1 (pregnan* or obstetric* or matern* or puerpe* or gestation*).ti,ab.

2 (antenatal* or ante-natal* or antepartum or ante-partum or prenatal* or pre-natal*).ti,ab.

3 (gravid* or nulligravid* or primigravid* or multigravid* or primipar* or multipar*).ti,ab.

4 Pregnancy/ or exp Gravidity/ or exp Parity/ or exp Parturition/ or exp "Pregnancy Maintenance"/ or exp "Pregnancy Outcome"/ or exp "Pregnancy, High-Risk"/ or exp "Pregnancy Complications"/

5 or/1-4

6 exp Smoking/

7 smok*.ti,ab.

8 or/6-7

9 5 and 8

10 Smoking Cessation/ or "Tobacco Use Cessation Devices"/

11 (smok* adj cessat*).ti,ab.

12 (nicotine replacement or ((nicotine or tobacco) adj2 (gum* or lozenge* or patch* or spray*)) or (pharmaco* adj2 (nicotine or tobacco or smoking)) or smoking cessation or incentivisation or incentive or voucher* or SMS or "text messag*" or "text-messag*" or "behavio$ support*" or counselling or vape or vaping or e-cigarette* or electronic cigarette* or inhalator* or (quit* adj (smok* or nicotine or tobacco))).ti,ab.

13 or/10-12

14 (couple*-based or couple* or partner* or spous* or family or families or wife* or husband* or girlfriend* or boyfriend* or house* or household*).ti,ab.

15 9 and 13 and 14

16 limit 15 to dc=20231204-20250514

**3. PsycInfo (ProQuest)**

(TI,AB(pregnan* OR obstetric* OR matern* OR puerpe* OR gestation* OR antenatal* OR ante-natal* OR antepartum OR ante-partum OR prenatal* OR pre-natal* OR gravid* OR nulligravid* OR primigravid* OR multigravid* OR primipar* OR multipar*) OR (MAINSUBJECT.EXACT.EXPLODE("Pregnancy") OR MAINSUBJECT.EXACT.EXPLODE("Pregnancy Outcomes"))) AND (TI,AB(smok*) OR MAINSUBJECT.EXACT.EXPLODE("Tobacco Smoking")) AND (MAINSUBJECT.EXACT.EXPLODE("Smoking Cessation") OR (TI,AB(smok* NEAR/2 cessat*) OR TI,AB(nicotine replacement OR ((nicotine OR tobacco) NEAR/2 (gum* OR lozenge* OR patch* OR spray*)) OR (pharmaco* NEAR/2 (nicotine OR tobacco OR smoking)) OR smoking cessation OR incentivisation OR incentive OR voucher* OR SMS OR "text messag*" OR "text-messag*" OR "behavio$ support*" OR counselling OR vape OR vaping OR e-cigarette* OR electronic cigarette* OR inhalator* OR (quit* adj (smok* OR nicotine OR tobacco))))) AND TI,AB(couple*-based OR couple* OR partner* OR spous* OR family OR families OR wife* OR husband* OR girlfriend* OR boyfriend* OR house* OR household*)

**4. Allied & Complementary Medicine - AMED (ProQuest)**

(TI,AB(pregnan* OR obstetric* OR matern* OR puerpe* OR gestation* OR antenatal* OR ante-natal* OR antepartum OR ante-partum OR prenatal* OR pre-natal* OR gravid* OR nulligravid* OR primigravid* OR multigravid* OR primipar* OR multipar*) OR (MAINSUBJECT.EXACT.EXPLODE("Pregnancy") OR MAINSUBJECT.EXACT.EXPLODE("Pregnancy Complications"))) AND (TI,AB(smok*) OR MAINSUBJECT.EXACT.EXPLODE("Smoking")) AND (MAINSUBJECT.EXACT.EXPLODE("Smoking Cessation") OR (TI,AB(smok* NEAR/2 cessat*) OR TI,AB(nicotine replacement OR ((nicotine OR tobacco) NEAR/2 (gum* OR lozenge* OR patch* OR spray*)) OR (pharmaco* NEAR/2 (nicotine OR tobacco OR smoking)) OR smoking cessation OR incentivisation OR incentive OR voucher* OR SMS OR "text messag*" OR "text-messag*" OR "behavio$ support*" OR counselling OR vape OR vaping OR e-cigarette* OR electronic cigarette* OR inhalator* OR (quit* adj (smok* OR nicotine OR tobacco))))) AND TI,AB(couple*-based OR couple* OR partner* OR spous* OR family OR families OR wife* OR husband* OR girlfriend* OR boyfriend* OR house* OR household*)

**5. British Nursing Index - BNI (ProQuest)**

(TI,AB(pregnan* OR obstetric* OR matern* OR puerpe* OR gestation* OR antenatal* OR ante-natal* OR antepartum OR ante-partum OR prenatal* OR pre-natal* OR gravid* OR nulligravid* OR primigravid* OR multigravid* OR primipar* OR multipar*) OR (MAINSUBJECT.EXACT("Pregnancy") OR MAINSUBJECT.EXACT("Pregnancy Complications"))) AND (TI,AB(smok*) OR MAINSUBJECT.EXACT("Smoking")) AND (MAINSUBJECT.EXACT("Smoking Cessation") OR (TI,AB(smok* NEAR/2 cessat*) OR TI,AB(nicotine replacement OR ((nicotine OR tobacco) NEAR/2 (gum* OR lozenge* OR patch* OR spray*)) OR (pharmaco* NEAR/2 (nicotine OR tobacco OR smoking)) OR smoking cessation OR incentivisation OR incentive OR voucher* OR SMS OR "text messag*" OR "text-messag*" OR "behavio$ support*" OR counselling OR vape OR vaping OR e-cigarette* OR electronic cigarette* OR inhalator* OR (quit* adj (smok* OR nicotine OR tobacco))))) AND TI,AB(couple*-based OR couple* OR partner* OR spous* OR family OR families OR wife* OR husband* OR girlfriend* OR boyfriend* OR house* OR household*)

**6. Cochrane Trials (Cochrane)**

#1 (pregnan* or obstetric* or matern* or puerpe* or gestation*):ti,ab,kw 110183

#2 (antenatal* or ante-natal* or antepartum or ante-partum or prenatal* or pre-natal*):ti,ab,kw 12977

#3 (gravid* or nulligravid* or primigravid* or multigravid* or primipar* or multipar*):ti,ab,kw 6931

#4 MeSH descriptor: [Pregnancy] explode all trees 31666

#5 #1 or #2 or #3 or #4 113698

#6 MeSH descriptor: [Smoking] explode all trees 9654

#7 (smok*):ti,ab,kw 44978

#8 #6 or #7 45012

#9 #5 and #8 3946

#10 MeSH descriptor: [Smoking Cessation] explode all trees 5674

#11 smok* NEAR cessat* 13011

#12 (nicotine replacement or ((nicotine or tobacco) NEAR/2 (gum* or lozenge* or patch* or spray*)) or (pharmaco* NEAR/2 (nicotine or tobacco or smoking)) or smoking cessation or incentivisation or incentive or voucher* or SMS or (text NEXT messag*) or text-messag* or (behavio$ NEXT support*) or counselling or vape or vaping or e-cigarette* or electronic cigarette* or inhalator* or (quit* NEAR/2 (smok* or nicotine or tobacco))):ti,ab,kw 49023

#13 #10 or #11 or #12 49376

#14 (couple*based or couple* or partner* or spous* or family or families or wife* or husband* or girlfriend* or boyfriend* or house* or household*):ti,ab,kw 87112

#15 #9 and #13 and #14
